# Perceptions of patients and health care professionals on postoperative pain management: Key factors influencing persistent opioid use

**DOI:** 10.1111/bjhp.70021

**Published:** 2025-09-11

**Authors:** Neetu Bansal, Rhiannon E. Hawkes, Li‐Chia Chen, Darren M. Ashcroft, Christopher J. Armitage

**Affiliations:** ^1^ Centre for Pharmacoepidemiology and Drug Safety, Drug Usage and Pharmacy Practice Group, Division of Pharmacy and Optometry, School of Health Sciences, Faculty of Biology, Medicine and Health University of Manchester, Manchester Academic Health Science Centre Manchester UK; ^2^ Manchester Centre for Health Psychology, School of Health Sciences University of Manchester Manchester UK; ^3^ Centre for Pharmacoepidemiology and Drug Safety, Division of Pharmacy and Optometry, School of Health Sciences, Faculty of Biology, Medicine and Health University of Manchester, Manchester Academic Health Science Centre Manchester UK; ^4^ NIHR Greater Manchester Patient Safety Translational Research Centre University of Manchester Manchester UK; ^5^ NIHR Greater Manchester Patient Safety Research Collaboration University of Manchester Manchester UK

**Keywords:** behaviour change techniques, experience‐based co‐design, persistent opioid use, post‐operative pain management

## Abstract

**Objective:**

This study applied the Theoretical Domains Framework (TDF) to explore the barriers and enablers to optimizing post‐operative pain management and supporting safe opioid use from the perspectives of both patients and health care professionals, applying the Theoretical Domains Framework (TDF).

**Design:**

Experience‐based co‐design (EBCD) qualitative study.

**Methods:**

In the initial phase of the EBCD approach, focus groups were conducted comprising 20 participants, including 8 patients and 12 health care professionals involved in post‐operative care. The data were systematically analysed using framework analysis and mapped to the TDF. Intervention functions and behaviour change techniques (BCTs) targeting each TDF domain were identified.

**Results:**

Analysis revealed significant barriers faced by patients, including a lack of continuity in care, inadequate preoperative preparation, and insufficient guidance on opioid tapering. Health care professionals reported barriers associated with deficiencies in knowledge, skills, and environmental resources, particularly workforce constraints and ineffective communication across care settings. Notable enablers included pharmacist‐led medication reviews and the adoption of digital technologies to enhance education and support tapering.

**Conclusions:**

This study identifies critical barriers and facilitators that influence postoperative pain management and opioid use. Targeted interventions are imperative to optimize these outcomes. Key recommendations include implementing pharmacist‐led medication reviews, integrating digital tapering support tools and enhancing preoperative educational efforts. Furthermore, strengthening communication pathways and addressing workforce challenges through dedicated training and resource allocation are essential. Future research should assess the efficacy of these tailored interventions across diverse clinical contexts to improve patient outcomes and refine prescribing practices.

**Statement of contribution**

**What is already known on this subject?**

- Post‐operative pain management and opioid use pose significant challenges in health care.
- Barriers to optimal opioid use include inadequate patient education, poor continuity in care and resource limitations for health care professionals.
- Theoretical frameworks like the TDF have been used to identify barriers and enablers in other contexts, but their application in post‐operative pain and opioid use is limited.

**What does this study add?**

- This novel study forms part of a wider study using the experience‐based co‐design (EBCD) methodology and reports the discovery phase, identifying key barriers to opioid tapering and post‐operative pain management from both patient and health care professional perspectives.
- It underscores the importance of continuity of care and pharmacist‐led reviews in effective opioid management.
- This study provides actionable recommendations for interventions, targeting patient education and health care professionals' capabilities, such as pharmacist‐led medication reviews, integrating digital support tools and improving pre‐operative education.

## INTRODUCTION

Post‐operative pain management remains a critical challenge in surgical care, particularly when balancing effective pain relief with the risks associated with opioid use (Gan, 2017). While opioids are commonly prescribed following surgery, their long‐term use carries significant risks, leading to dependence, misuse and adverse health outcomes (Myles & Bui, 2022). It has been estimated that approximately 6%–10% of opioid‐naïve surgical patients become long‐term opioid users, highlighting the need for careful post‐operative opioid management (Brummett et al., 2017; Soneji et al., 2016).

Beyond health implications, opioid‐related complications impose a substantial economic burden (Friebel et al., 2022). In the United Kingdom (UK), opioid dependence and misuse contribute significantly to increased health care costs for the National Health Service (NHS) due to hospital readmissions, prolonged recovery times and needs for addiction treatment services (Roberts & Richards, 2023). Opioid misuse also poses a profound societal impact, leading to reduced productivity and lower quality of life. Addressing these challenges requires an understanding of the perspectives of both patients and health care professionals regarding post‐operative opioid use (Cho et al., 2021; Walton et al., 2023). Patients' expectations for pain relief are often shaped by pre‐operative experiences, while health care professionals' prescribing decisions are influenced by clinical guidelines, perceived patient needs and concerns about misuse (Varrassi et al., 2023). Misalignments in expectations and ineffective communication between patients and providers can complicate opioid tapering and post‐operative pain management (Klueh et al., 2019).

Various strategies have been implemented to reduce opioid use following surgery, such as enhanced recovery after surgery (ERAS) protocols, multimodal analgesia and non‐opioid pain management strategies. These methods have shown promise in reducing opioid requirements without compromising pain relief (Simpson et al., 2019). However, challenges remain, such as inconsistent adherence to ERAS protocols, excessive opioid prescribing at discharge and gaps in follow‐up care (Ahmed et al., 2012; Collaborative, 2024).

Further research is needed to develop tailored interventions that address barriers to opioid reduction, aligning patient‐provider expectations, improving communication and integrating behavioural strategies for opioid tapering. Addressing these gaps can help optimize pain management while minimizing the risks of long‐term opioid use. The Theoretical Domains Framework (TDF) provides a structured approach to exploring cognitive, emotional, and environmental influences on behaviour. It has been widely applied to understand behaviour change in health care (Keyworth et al., 2019) and is used in this study to explore the barriers and enablers of opioid prescribing and use. Therefore, this study seeks to understand the perceptions of both patients and health care professionals regarding post‐operative pain management and opioid use, with the goal of improving practices and reducing opioid‐related harms.

## METHODS

### Design

We conducted a qualitative study utilizing the Experience‐Based Co‐Design (EBCD) approach, engaging both patients with first‐hand experience of being prescribed opioids post‐surgery and health care professionals working within the UK's NHS (Bate & Robert, 2006). EBCD is a participatory methodology that brings together patients and health care staff to co‐design improvements in health care services by first exploring their experiences and then collaboratively developing solutions (Bate & Robert, 2006). The EBCD process involves an initial discovery phase, where patients and health care professionals participate in separate focus groups or interviews to share their experiences and identify key issues, followed by joint co‐design workshops where both groups work together to prioritize and design intervention components (Donetto et al., 2015).

This paper reports findings from the discovery phase, comprising separate focus groups with patients and health care professionals to explore barriers and enablers to optimizing post‐operative opioid use. The subsequent co‐design workshops, where both groups worked together to develop the intervention to identify patients at risk of long‐term opioid use, are reported in a separate manuscript. Conducting focus groups separately allowed participants to speak freely and ensured that the distinct perspectives of each group were captured before coming together to design solutions collaboratively.

### Theoretical framework

We applied the Capability, Opportunity and Motivation‐Behaviour (COM‐B) model, which includes physical capability, psychological capability, physical opportunity, social opportunity, reflective motivation and automatic motivation to inform the interview questions (Michie et al., 2011). This framework was linked to the TDF for subsequent analysis (Appendix S1). The TDF facilitates a thorough examination of environmental, social, cognitive and emotional factors influencing behaviours, making it suitable for identifying barriers and enablers in this context (Atkins et al., 2017). Behaviour Change Techniques (BCTs) were extrapolated from the TDF domains using the Theories and Techniques Tool (https://theoryandtechniquetool.humanbehaviourchange.org/tool), ensuring the findings were grounded in evidence‐based behaviour change strategies (Johnston et al., 2020).

### Participants

Eligible participants included adults over 18 years who self‐reported having undergone a surgical procedure and had been prescribed opioids for post‐operative pain management. Health care professionals included experts with experience in managing post‐operative pain within the UK's NHS. To capture diverse perspectives, participants were selected from various professional backgrounds and care settings. All participants were provided with study information and gave written informed consent prior to participation.

### Recruitment

Patients were recruited through social media advertisements (Twitter/X, LinkedIn and Facebook) and partnership with pain charities, such as ‘Pain Concern’ which disseminated study advertisements via their social media platforms and website. Health care professionals were invited through professional networks, social media and snowball sampling. Written informed consent was obtained from all participants prior to their involvement.

### Procedures

Data collection took place between March 2024 and July 2024. The patient focus group was conducted in person to ensure participants felt supported and comfortable discussing sensitive and potentially distressing experiences related to pain and opioid use, with immediate access to in‐room support if needed. In contrast, the health care professional focus group was conducted online to accommodate busy clinical schedules and enable participation from a wider geographical area. The lead researcher (NB) and project supervisor (LCC) facilitated the focus groups with the other two researchers (WCL and FA).

Upon arrival or logging in, participants were welcomed and provided with a detailed briefing on the purpose of the study and the structure of the focus group sessions. Facilitators introduced the session by outlining the objectives of the discussion and establishing ground rules to foster a supportive, open and respectful environment for dialogue. Participants were informed about the confidentiality and anonymity of their contributions, and consent was obtained for the recording of the sessions using an encrypted audio recorder.

Each focus group session lasted approximately 120 min. The sessions were audio‐recorded and transcribed verbatim by a third‐party approved transcription company, which had signed confidentiality agreements to ensure data security. All transcripts were pseudonymised before analysis.

### Materials

Facilitators used topic guides (Appendices S2 and S3) that were informed by the COM‐B model (Michie et al., 2011). These guides ensured focused discussions while allowing flexibility for participants to share their insights.

### Analysis

A Framework analysis approach (Gale et al., 2013) was used to analyse the data structured around the TDF. Framework Analysis was selected as it enables systematic coding of qualitative data into pre‐defined domains while allowing codes to be incorporated, providing a transparent and structured approach suited to implementation research. The lead researcher (NB) undertook inductive open coding to identify codes and themes grounded in participants' experiences. These codes were discussed with a second researcher (RH). Following the inductive coding, a deductive approach was applied whereby key themes were mapped to the relevant TDF by two researchers (NB and RH) to identify theoretical constructs underpinning participants' views. Themes and coding decisions were discussed with a third author (CJA).

Microsoft Excel was used to develop the coding framework, utilizing principles of the TDF approach to map the data to relevant theoretical domains. This approach was chosen to enable thorough explorations of both predetermined and emergent issues whilst using the TDF as an explanatory framework. Each theoretical domain of TDF encompasses various aspects of behaviour, such as knowledge, skills, emotions, and environmental influences, providing a structured approach to identify the psychological and contextual factors affecting individuals' decisions and actions (Cane et al., 2012).

Overall, the TDF synthesizes 33 behaviour theories into 14 domains, designed to help researchers understand and influence behaviour change in health and clinical settings (Table 1). The frequency of TDF domains was used to complement the qualitative thematic interpretation by indicating areas of emphasis across participant groups. BCTs were subsequently extracted from the identified theoretical domains using the Theories and Techniques Tool (Johnston et al., 2020). This tool is an evidence‐based resource that maps links between theoretical domains and specific BCTs, providing a systematic approach to selecting techniques most likely to address identified barriers and enablers (Johnston et al., 2020). The identified BCTs were then used to guide the design of an intervention during subsequent co‐design sessions (reported separately), ensuring that the intervention was both theoretically grounded and practically relevant.

**TABLE 1:** Coding for theoretical domains framework.

Domain	Explanation
Knowledge	An awareness of the existence of something
Skills	An ability or proficiency acquired through practice
Social/professional role and identity	A coherent set of behaviours and displayed personal qualities of an individual in a social or work setting
Beliefs about capabilities	Acceptance of the truth, reality or validity about an ability, talent or facility that a person can put to constructive use
Optimism	The confidence that things will happen for the best or that desired goals will be attained
Beliefs about consequences	Acceptance of the truth, reality or validity about outcomes of a behaviour in a given situation
Reinforcement	Increasing the probability of a response by arranging a dependent relationship or contingency, between the response and a given stimulus
Intentions	A conscious decision to perform a behaviour or a resolve to act in a certain way
Goals	Mental representations of outcomes or end states that an individual wants to achieve
Memory, attention and decision processes	The ability to retain information, focus selectively on aspects of the environment and choose between two or more alternative
Environmental context and resources	Any circumstance of a person's situation or environment that discourages or encourages the development of skills and abilities, independence, social competence and adaptive behaviour
Social influences	Those interpersonal processes that can cause individuals to change their thoughts, feelings, or behaviours
Emotion	A complex reaction pattern, involving experiential, behavioural and physiological elements, by which the individual attempts to deal with a personally significant matter or event
Behavioural regulation	Anything aimed at managing or changing objectively observed or measured actions

### Researcher positioning

The lead researcher (NB) is a surgical pharmacist with extensive experience in post‐operative pain management and opioid prescribing. While this expertise provided valuable insights into the research topic, it may also have influenced the interpretation of findings. To mitigate potential biases and ensure a balanced and transparent approach, a multidisciplinary team, including a psychologist (CJA) and a researcher with a background in health psychology (RH), was involved in the analysis and interpretation.

### Ethical approval

Ethical approval for this study was obtained from the University of Manchester research and ethics committee (UREC 2024‐17993‐32933). Each participant gave full informed consent prior to participating.

## RESULTS

### Participant demographics

We recruited 8 patients with a history of having been prescribed opioids for post‐operative pain management following surgery. Participants were aged 25–84 years. The most reported age range was 45–54 years (*n* = 4). The median age, estimated based on range midpoints, was approximately 49.5 years (Table 2). The majority of patient participants were British English (*n* = 5), with the remainder identifying as British Asian/Asian and one as British African/African (*n* = 3).

**TABLE 2:** Characteristics of eight patient participants.

Gender	Ethnicity	Age range	Types of surgery undergone
Female	British English	45–54	Spinal/abdominal surgeries/upper GI surgery
Female	British English	65–74	Orthopaedic surgery
Male	British African/African	25–34	Orthopaedic surgery
Male	British English	45–54	Cardiac surgery
Male	British Asian/Asian	45–54	Orthopaedic surgery
Female	British English	65–74	Colorectal/Orthopaedic surgery
Female	British English	75–84	Orthopaedic surgery
Female	British Asian/Asian	45–54	Orthopaedic surgery

The health care professional participants (*n* = 12) represented a diverse group in terms of professional roles, years of experience and workplace settings. Most participants were pharmacists (*n* = 9), with roles ranging from medicines optimization pharmacists to surgical pharmacists working across academic institutions, NHS hospitals and Clinical Commissioning Groups. Additionally, three participants were doctors, including two academic general practitioners (GPs) and one consultant anaesthetist with a special interest in pain.

Health care professional participants were predominantly White British (*n* = 9), with the remaining identified as British Asian/Asian (*n* = 3). The group was evenly distributed across age ranges, with most participants aged between 35 and 54 years. A significant proportion of participants (*n* = 9) had over 20 years of experience in their respective fields, reflecting a high level of expertise (Table 3).

**TABLE 3:** Characteristics of 12 health care professional participants.

Gender	Ethnicity	Age range	Position	Profession	Employer	Years in current profession
Male	White British	45–54	Professor in clinical pharmacy practice and specialist pharmacist in pain management	Pharmacist	Academic institution	>20 years
Female	White British	35–44	Advanced pharmacist practitioner in pain	Pharmacist	General practice	>20 years
Female	White British	55–64	Senior medicines optimization pharmacist	Pharmacist	Clinical commissioning group	>20 years
Male	British Asian	35–44	Academic GP	Doctor	Academic institution/general practice	5–10 years
Female	White British	25–34	Orthopaedic pharmacist	Pharmacist	NHS Hospital	2–5 years
Female	White British	45–54	Medicines safety lead pharmacist	Pharmacist	Academic institution	>20 years
Female	White British	35–44	Academic GP	Doctor	Academic institution/general practice	5–10 years
Male	British Asian/Asian	45–54	Consultant anaesthetist with an interest in pain	Doctor	NHS Hospital	>20 years
Male	White British	25–34	Senior medicines optimization pharmacist	Pharmacist	Clinical commissioning group	2–5 years
Female	White British	45–54	Head of medicines optimization and governance	Pharmacist	NHS hospital	>20 years
Male	British Asian/Asian	45–54	Deputy chief pharmacist, chair of the hospital pharmacy pain group	Pharmacist	NHS hospital	>20 years
Male	White British	45–54	Head of medicines optimization and prescribing	Pharmacist	Clinical commissioning group	>20 years

To present participant quotes, each is denoted with a letter and a number (e.g., R1, R2, R3). The letter ‘R’ represents the role of the participant (e.g., P for patient or H for health care professional), and the number identifies the individual participant within their group. This approach ensures confidentiality while allowing the reader to distinguish between participants and track patterns in the data.

The key themes identified from patients and health care professionals' focus groups are summarized as follows (Table 4).

**TABLE 4:** Summary of patient and health care professionals' views.

Domain	Theme	Patients	Health care professional
Environmental context and resources	Continuity of care and health care interactions	Lack of continuity and personalized care; need for improved pre‐operative preparation	Workforce constraints, unclear responsibilities, fragmented communication during transitions of care
	Use of digital technology and pharmacist‐led reviews	Pharmacist‐led reviews highly valued, positive experiences with digital and non‐pharmacological methods	Digital tools (electronic records, digital education) useful for consistent care, good patient–professional relationships crucial
Knowledge	Communication gaps and education needs	Insufficient and inconsistent communication across care interfaces; fragmented care	Need improved clarity and consistency of communication about pain management and opioid tapering
Emotion	Psychological impact and coping strategies	Emotional burden of pain, anxiety about opioid use, reliance on self‐taught coping strategies	N/A
Skills	Pain management challenges	N/A	Complexity in managing acute pain in chronic pain patients; remote consultations hinder comprehensive assessment
	Skills development gaps	N/A	Lack of structured training and guidance for opioid tapering; insufficient skills among non‐specialists

### Results from patient focus group

Three key theoretical domains were identified that explained the barriers and enablers to effective post‐operative pain management: environmental context and resources (42/101 occurrences), knowledge (14/101 occurrences) and emotion (13/101 occurrences).

#### Environmental context and resource domain

Three barriers and one enabler were described in relation to this domain. Patients described their interactions with health care professionals and the health care system, difficulties with managing acute pain in patients with chronic and co‐morbid issues as well as inadequate pain management post‐surgery.

##### Health care system and provider interactions (barrier)

Patients described a lack of continuity of care and unclear responsibility across care transitions. Experience with pain clinics was often poor due to strict adherence to guidelines and protocols in hospitals resulting in a lack of individualized care. Patients also expressed a need for personalized care plans and improved pre‐operative preparation. Positive interactions that served as enablers included pharmacist‐led reviews in primary care, which were perceived as valuable, as well as positive experiences with non‐pharmacological approaches, such as acupuncture and cognitive behaviour therapy.

P2 (Female, 68 years) described her distress after her pain management regime was changed without consultation:

But the anaesthetist in his wisdom decided to change my regime, post‐op, from my knee surgery. So, there I was, the second night, screaming the place down, when other people were, you know, quietly sleeping.

P5 (male, 48 years) responded by sharing how his pain management had been handled differently, though still not ideally:

No, no, for me it's different because I'm…it was…I…it's through a pain clinic that I have my, you know, annual…every nine months, the pain clinic reviews it and, you know, they just kept upping the dose every time.

##### Chronic pain and co‐morbid issues (barrier)

Participants noted that health care professionals often overlooked co‐existing comorbidities when managing post‐operative pain, leading to insufficient advice or support for managing pain after surgery. Patients felt their autonomy was disregarded, with limited involvement in decisions regarding their care. The complexity of managing acute pain superimposed on chronic pain posed considerable challenges, resulting in common undertreatment and inadequate pain relief.

P6 (female, 67 years) shared her frustration:

And I don't think it's got through to the MSK [musculoskeletal] people that they're treating a rheumatoid who's in pain constantly. Because you've always got some pain, you know.

P2 (female, 68 years) responded, agreeing and highlighting how this affected her pain management after surgery:

Also, when you come out of surgery, they tend to forget that your tolerance to drugs is significantly higher. I mean, in my case, the amount I was taking, I mean, my doctor said he would be comatose if he took even one of my doses a day, you know, the morning dose is so powerful. (P2, female, 68 years)

##### Inadequate pain management post‐surgery (barrier)

Patients described inadequate pain management after surgery, highlighting several key issues, such as a lack of education on tapering opioids, recognising and managing withdrawal symptoms, understanding potential side effects and effectively addressing post‐operative pain. Additionally, there was significant variation in opioid quantities provided upon discharge, with some patients feeling they received insufficient supplies, while others were discharged with excess amounts, resulting in unused opioids being left at home.

P4 (male, 52 years) emphasized the need for better tapering advice:

That's part of the problem with, when people are discharged from hospital, is that they're discharged with a whole load of opioids that says, take this three times a day. And, actually, it needs to say, don't stop this suddenly, because otherwise you'll get whole load of side‐effects. But it should be, after you've taken this for a week, after you've been home for a few days, when you feel ready, start to drop the dose, just take half the dose, and then, and actually talk about proper tapering.

P7 (female, 77 years) agreed, adding her own experience of inadequate information:

Basically, I got my medication, they gave me morphine and Laxido, and that's it, no information in regard to how often to take it, what it contains or possible side effects.

##### Pharmacist‐led reviews (enabler)

Participants highlighted the value of pharmacist‐led reviews for supporting opioid reduction following surgery. Many reported positive experiences, citing the pharmacists' expertise in medication management, accessibility and patient‐centred approach. Pharmacists were seen as instrumental in facilitating safe and effective opioid tapering, offering tailored guidance and monitoring to address individual patient needs. These reviews were also credited with improving communication between patients and health care teams, fostering trust and promoting shared decision‐making.

And I'm working, I've been working for a year with the pharmacist at the practice, on a reduction programme. And we've got rid of the tramadol and reduced the MST [Morphine sulphate], the slow morphine…It needs education and monitoring, and GPs need time to do that. And GPs don't have time. It's the pharmacist I've been working with on that, though. Well, the pharmacist, yeah, absolutely, yeah, yeah, they're brilliant. (P2, female, 68 years)

#### Emotion domain

One barrier was identified regarding the psychological impact of pain and the challenges in coping mechanisms. Patients expressed how the emotional toll of pain, combined with insufficient support, often hindered their ability to effectively manage their recovery.

##### Psychological impact and coping mechanisms (barrier)

Patients conveyed the psychological burden of pain and various coping strategies they developed over time. Some expressed anxiety about using medications, particularly opioids, prompting exploration of non‐pharmacological alternatives, such as mindfulness or other self‐care strategies. In the absence of professional guidance, patients often relied on self‐taught methods to manage their pain, gradually becoming ‘experts’ in addressing their own pain. This sense of self‐reliance, while empowering for some, underscored gaps in the health care system's ability to provide consistent support.

In other words, work with people, but understand that they'll have a level of expertise about their own condition. It's how you deal with that, because you're defining… all three of you, would seem to me to be expert patients. (P8, female, 54 years)

#### Knowledge domain

Communication was identified as a significant barrier in this domain. Patients and carers reported challenges in receiving clear and consistent information from health care professionals. Additionally, poor communication across care interfaces impeded effective pain management.

##### Communication (barrier)

Patients highlighted the lack of effective communication as a significant barrier. They reported insufficient engagement from health care professionals both to themselves and their carers, leaving them feeling uninformed and unsupported. Poor communication across care interfaces exacerbated fragmented care. Participants emphasized the need for a multidisciplinary approach to improve communication and ensure a more coordinated and patient‐centred experience in managing post‐operative pain.

P4 (male, 52 years) shared his frustration about the lack of proactive communication regarding side effects:

There's a little bit more, which is, no one at any time has ever talked to me about opioids induced constipation. GPs deal with constipation when it happens, rather than proactively preparing you for it, and saying, you need to do that.

P1 (female, 54 years) responded, adding her concerns about poor dialogue with GPs:

Well, I think…I know there's a lot more restrictions have come in lately for the GPs, which is a good thing. But they need…they've got no dialogue with the patients. They have no dialogue with the patients about the opioid use or anything. You know, they're not interested, they just, like I say, dish them out, right, that's her done. She won't be moaning this month. We've done a prescription.

#### Behaviour change techniques identified from the patient focus group

The BCTs identified from the patient focus group included providing social support (unspecified) such as support from GPs or self‐help groups, and social support (practical). Other BCTs included instructions to patients on how to perform the behaviour (e.g., how to taper off opioids), providing information on health consequences, restructuring the physical environment, reducing negative emotions such as anxiety or fear of opioid withdrawal and using problem‐solving strategies, such as health monitors to manage and cope with pain. To further clarify how these BCTs might be implemented, Table 5 provides examples of delivery strategies for each BCT.

**TABLE 5:** Behaviour change techniques identified from the focus groups.

Behaviour change technique	Example of delivery to patients
Patients' focus group	
2.4. Self‐monitoring of behaviours	Introducing health monitoring tools (e.g., pain trackers or wearable devices) to help patients track progress and manage pain
3.2. Social support (practical)	Providing direct assistance, such as scheduling medication reviews or arranging pharmacy‐led tapering consultations
4.1. Instructions on how to perform the behaviour	Step‐by‐step guidance on how to safely taper opioid use, including personalized tapering plans and demonstrations
5.1. Information about health consequences	Leaflets or videos explaining the long‐term risks of opioid use, such as dependence and benefits of reducing usage
11.2. Reduce negative emotions	Providing psychological support, such as mindfulness or stress management resources, to alleviate anxiety or fear
12.1. Restructuring the physical environment	Offering reminders via apps to help patients manage medication adherence effectively
Health care professionals' focus group	
4.1. Instructions on how to perform a behaviour	Providing detailed guidance and training on how to taper opioids effectively, including step‐by‐step instructions on reducing doses gradually and safely
5.1. Information about health consequences	Educating prescribers that opioids are intended for short‐term use only in the post‐operative context. This enables them to inform patients about the risks of long‐term use and set appropriate expectations for opioid therapy
7.1. Prompts or cues	Integrating alerts or reminders into electronic health care records to prompt prescribers to assess opioid prescriptions and determine if tapering is appropriate
8.7. Graded tasks	Setting clear expectations with patients early on, such as explaining that opioid use will be short‐term and tapering will occur within a specific timeframe. Breaking the process into manageable steps helps patients and professionals track progress

*Note*: The hierarchical structure number was listed according to the Behaviour Change Technique Taxonomy version 1 (BCTTV1).

### Results from health care professional focus group

In the health care professional focus group, three key theoretical domains were identified concerning barriers and enablers to effective post‐operative pain management: environmental context and resources (54/124 occurrences), knowledge (16/124 occurrences) and skills (38/124 occurrences).

#### Environmental context and resources domain

Two barriers and two enablers were described in relation to this domain. Barriers included health care system and provider interactions, communication and continuity of care. In contrast, enablers encompassed the use of digital technology, such as electronic health records and digital pain management tools and strong patient and health care professional relationships.

##### Health care system and provider interactions (barrier)

Workforce pressures within the UK NHS system, with limited resources and staff shortages, significantly affected post‐operative pain management delivery, causing ambiguity in responsibilities for pain management during care transitions. Systemic variation in practices, including differences in how hospital discharge reviews were conducted, was also highlighted as a barrier. Inconsistent use of guidelines and protocols contributed to variability in care, leading to a lack of standardised approaches to post‐operative pain management.

And obviously coupled in with this the workforce pressures that primary care is facing, you don't seem to see the same GP twice or, you know, sometimes it's not a GP, it's a pharmacist or a nurse or something. And that just adds to the inertia where continuation just happens. (H10)

##### Communication and continuity of care (barrier)

Barriers related to communication and continuity of care were identified as significant challenges in post‐operative pain management. Inadequate communication across care interfaces led to fragmented care and confusion about roles and responsibilities. Moreover, inadequate communication with patients and insufficient provision of unclear information about managing pain post‐surgery were also highlighted. Furthermore, the absence of a biopsychosocial approach to pain management, one that integrates biological, psychological and social factors, was noted as a critical gap (Kovačević et al., 2024). Health care professionals emphasized the need for holistic and individualized care to address the complex nature of post‐operative pain effectively.

The top of my list was communication, and you've got two work systems really that you're communicating between, between secondary care and primary care. And the two work systems are very different in most places in the country…So how that communication happens and how timely it is and how clear we are about how long we're expecting the pain to last for. Communication for patients, acknowledging that they are not going to remember what they're told in a hospital, and they need something to refer to afterwards. And that there should be one consistent message across a system as well. (H4)

##### Use of digital technology (enabler)

Technological advancements were identified as a key enabler in improving post‐operative pain management. Health care professionals highlighted the potential for innovative approaches, such as providing patients with instructional videos at discharge instead of paper leaflets. These videos could explain the risks and benefits of opioids and offer tapering advice in a more engaging and accessible format.

Efforts to improve inclusivity were also noted, including the provision of informational leaflets in multiple languages and the use of QR codes to grant patients easy access to digital resources. Additionally, electronic health care records and prescribing indicators were recognised as valuable tools to identify patients discharged on opioids, facilitating timely interventions and follow‐up care.

But also, as we're moving into more digital technology, something that they can watch on discharge that explains about their medicines that can be translated into many languages is probably something that we should be trying to do. (H7)

##### Patient–health care professional relationship (enabler)

Trust and rapport in the patient‐health care professional relationship were considered essential for successful transitions to alternative pain management strategies. Health care professionals highlighted the importance of clear and early communication to set realistic expectations about opioid use, such as informing patients that opioids are not typically required beyond a specific timeframe. These early conversations helped align patients' expectations and reinforced the rationale for tapering opioids.

Continuity in communication across consultations was also noted as beneficial, as it provided a reference point to build on patients' existing understanding and expectations. Structured pain reviews were seen as valuable opportunities to explore the reasons for discontinuing opioids, helping patients to understand the benefits and remain motivated to engage in their pain management plan.

And having that…so then when they come back again, you know, there's something, a reference point in saying we had a conversation, these are the risks, and continuing it makes it much easier. Their expectations are already there in their head that there's a reason we're not going to be continuing it. (H4)

#### Knowledge domain

Education and awareness were identified as key barriers within this domain, highlighting gaps in understanding and dissemination of information related to effective post‐operative pain management and opioid prescribing practices.

##### Education and awareness (barrier)

A knowledge gap exists in opioid prescribing practices, specifically around the short‐term use of opioids after surgery. There is a clear need for education for both health care professionals and patients to emphasize the importance of using opioids for the shortest duration possible. A common challenge identified was the lack of clear guidance on tapering off opioids, particularly the absence of definitive timelines for weaning patients off them. Although efforts are being made to encourage GPs to aim for a gradual weaning approach, the effectiveness of this approach remains unclear without specific timelines. Therefore, education is needed on both fronts: primary care needs improved guidance on opioid tapering, while secondary care requires more robust education on post‐surgical pain management and the importance of setting clear expectations.

Additionally, certain patient groups, such as those who may be housebound after discharge, present a significant challenge. Many of these patients depend on remote assessments, complicating the accurate evaluation of pain levels compared to in‐person consultations. During face‐to‐face visits, clinicians can visually assess a patient's level of pain and discomfort, but remote consultations, such as those conducted over the phone, may result in missed cues and hinder accurate assessment.

And I think a lot of is around education both for patients and for GPs. Not only GPs but other non‐pain‐based prescribers around, you know, the fact that it is short term. And I think we've not been very, very good with that short term piece. (H10)

#### Skills domain

Challenges within this domain highlighted the need for skill development among health care professionals, with enablers including the availability of non‐pharmacological alternatives. Many clinicians, particularly those in non‐specialist roles such as GPs, may lack the necessary skills to manage post‐surgical pain effectively and to understand the complexities of opioid prescribing. This gap in skills is often compounded by limited training in recognizing when patients need opioid tapering or alternative pain management strategies.

Moreover, participants discussed that many health care professionals may not be adequately equipped to conduct comprehensive assessments. To address these issues, they suggested a clear need for enhanced pain management training across both primary and secondary care settings, ensuring health care professionals can provide optimal care from initial pain management to opioid discontinuation.

##### Challenges in pain management (barrier)

Identified gaps pertained to managing complex cases, particularly for patients with chronic pain and already on long‐term opioids prior to surgery. Setting realistic expectations for patients is crucial, as misaligned expectations can complicate post‐surgery opioid tapering. Patients often fear losing functionality or experiencing unmanaged pain without opioids, complicating the tapering process.

I guess, what I was just reflecting is that I think the skills around acknowledging when it's moving from acute pain to chronic non‐cancer pain and that the step change has to happen then in how we manage it. Because it's almost a different thing, isn't it? (H4)

##### Skills development (barrier)

A significant barrier identified was the lack of structured opportunities for health care professionals to develop the essential skills in pain management and opioid deprescribing. Many clinicians expressed difficulty in managing complex cases, such as patients already on long‐term opioids before surgery. This gap in skills often leads to inconsistent tapering practices and an over‐reliance on opioids due to unfamiliarity with alternative pain management strategies.

Time constraints further obstructed skills development, as tapering plans and consultations often require thorough discussions to address patient fears about loss of function or unmanaged pain without opioids. Participants emphasized the urgent need for targeted training programmes and structured guidance in opioid tapering, non‐pharmacological alternatives and effective communication strategies to set realistic patient expectations. Without addressing these barriers, health care professionals will struggle to deliver optimal care.

So I would agree that some more education in…for GP practice clinicians, not…because a lot of the time, it is now practice pharmacists that are reviewing and dealing with this medication. So, I think they would all welcome more information, more guidance because I think it is an area that it's a bit…as you say, it is quite patient‐centred, and it's going to vary, but I think if there was some more…some stronger messaging as to realistically how long we would expect them to be continued for, just for some guidance. (H8)

#### BCTs identified from the theoretical domains from the health care professional focus group

Four key BCTs were identified from the theoretical domains in the health care professional focus group. These include providing instructions on how to perform a behaviour, such as guiding health care professionals on how to structure and communicate tapering plans with patients, and information about health consequences, including educating patients about the risks of long‐term opioid use and the benefits of tapering. These strategies were particularly important in supporting informed, shared decision‐making and increasing patient engagement. In addition, providing prompts and cues and graded tasks were also identified as crucial BCTs. These included the incorporation of alerts or reminders in electronic health care records to prompt health care professionals to evaluate opioid prescriptions and consider tapering based on the patient's progress. Graded tasks involving setting clear expectations with patients regarding short‐term use of opioids and outlining a gradually tapering plan over a specified timeline (several weeks) can make the process more manageable. By establishing these expectations early, patients are better prepared for the gradual reduction and can mentally and physically adjust to the changes. Table 5 outlines these BCTs along with examples of how they might be delivered in practice.

## DISCUSSION

### Main findings

This is the first study using the TDF (Atkins et al., 2017) to identify the barriers and enablers for both patients and health care professionals regarding post‐operative pain management and opioid use. The findings highlight the complexities of managing post‐operative pain while addressing the risks associated with long‐term opioid use, underscoring the need for interventions that align patient and provider expectations.

This research makes two key contributions to the existing literature. Firstly, it identifies the three most prominent TDF domains for both patients and health care professionals, offering valuable insights into the factors that most significantly influence effective post‐operative pain management. Secondly, it provides actionable recommendations on the intervention functions and BCTs that should be incorporated into future interventions to optimize pain management and promote safe opioid tapering.

For patients, key challenges were linked to the domains of environmental context and resources, emotion and knowledge. Significant barriers included a lack of continuity in care, limited pre‐operative preparation and insufficient guidance on opioid tapering. These findings resonate with previous research demonstrating the fragmented nature of pain management across care transitions and the variability in discharge opioid prescribing practices (Jordan et al., 2021; Klueh et al., 2019; Kuntz et al., 2020). Despite these challenges, pharmacist‐led reviews were identified as a significant enabler, demonstrating pharmacists' potential in bridging communication gaps, providing personalized tapering guidance and improving patient outcomes.

Among health care professionals, barriers were primarily related to environmental context and resources, knowledge and skills. Resource constraints, workforce pressures and inadequate communication between hospital and community care settings were major obstacles to effective pain management. These findings align with prior studies that emphasized the importance of multidisciplinary teamwork and continuity of care to overcome systemic challenges (Hinds et al., 2022). Enablers such as the use of digital technology, including electronic health record alerts and instructional videos, were regarded as promising strategies to support opioid tapering and enhance patient education. For instance, one study explored the use of digital and mobile health services, finding that digital videos and text messaging interventions can enhance patient education and provide continuous support throughout the tapering process (Magee et al., 2022).

The findings of this study align closely with Keyworth et al. (2019), who used the TDF to explore barriers and enablers for health care professionals delivering opportunistic behaviour change interventions during routine consultations. Similar to Keyworth et al., our study found that health care professionals recognized the value of behaviour change interventions but faced barriers such as workload, scepticism about capabilities and environmental constraints. While Keyworth et al. focused on opportunistic interventions, our study expanded these insights to the context of post‐operative pain management, addressing the perspectives of both patients and providers and broadening the scope of identified intervention strategies.

This study identified several BCTs to address barriers and enhance enablers. For patients, interventions should focus on providing social support (enhancing *social opportunity*), delivering clear information on the health consequences of opioid use (increasing *psychological capability* and *reflective motivation*), and promoting problem‐solving strategies for managing pain (enhancing *psychological capability* and *reflective motivation*). For health care professionals, the use of graded tasks and detailed guidance on how to taper opioids effectively (building *psychological capability*), as well as prompts or cues (supporting *physical opportunity*) was seen as critical. These findings align with the Behaviour Change Wheel framework, which emphasizes the importance of tailoring interventions to address capability, opportunity and motivation (Michie et al., 2011).

### Implications for practice and implementation

The findings of this study have significant implications for clinical practice. Improving continuity of care is essential, with structured handovers and follow‐up systems to ensure seamless transitions from hospital to community settings. Pharmacists can play a more central role in post‐operative pain management by conducting regular medication reviews and supporting opioid tapering. Digital tools such as electronic alerts to clinicians on patients still on opioids post‐surgery, QR codes for patients providing information on the risks of ongoing opioid use and multilingual patient information instructional videos can standardize and improve access to guidance for both patients and health care professionals.

A recent systematic review identified key BCTs effective in opioid deprescribing interventions, including behavioural instructions, behaviour substitution, outcome goal setting and social support (Bansal et al., 2024). These BCTs are summarized in Appendix S4. These findings align with our recommendations to incorporate clear guidance, alternative pain management strategies and enhanced support systems in post‐operative care. Integrating these BCTs into interventions may help address identified barriers and leverage enablers to optimize pain management and facilitate safe opioid tapering. Additionally, patient education should be prioritized, with a focus on providing clear, tailored information about opioid use, tapering strategies and alternative pain management approaches.

There is an urgent need for effective interventions to optimize opioid use following surgery, particularly across care transitions. While some interventions exist, our recent systematic review identified that many are limited in scope and lack a robust theoretical underpinning based on established behavioural science frameworks such as the TDF or BCT taxonomy. This limits their reproducibility, scalability and potential for sustained impact in real‐world settings.

To address this gap, the current programme of work adopted an EBCD approach, ensuring that the developed intervention was grounded in the lived experiences of both patients and health care professionals. The co‐design process was facilitated by conducting two separate focus groups with patients and health care professionals before conducting joint co‐design workshops which are reported in a separate paper. These workshops not only captured critical insights into barriers and enablers of opioid tapering but also enabled collaborative design of intervention components that are acceptable, feasible and theory informed.

### Strengths and limitations

A key strength of this study is the use of the TDF, as it provided a comprehensive approach for identifying barriers and enablers across multiple domains. This theoretical grounding was crucial to the EBCD process conducted in a separate paper, as it helped ensure that insights captured during the focus groups were not only rooted in lived experiences but also aligned with established behavioural science principles. Additionally, the inclusion of diverse participants, including patients and health care professionals, offered a holistic understanding of the challenges and opportunities in post‐operative pain management. While participants were diverse in terms of age, gender and surgical experiences, data on socio‐economic status or education levels were not collected. Given the relationship between health inequalities and opioid use (Atkins & Mukhida, 2022), this represents a limitation of the study as such factors may influence pain management experiences and outcomes. Furthermore, this study was limited by its focus on the UK health care context, which may limit the generalizability of the findings to other settings globally. Future studies should examine whether the findings hold across different health care systems and cultural contexts.

## CONCLUSION

This study provides valuable insights into the barriers and enablers influencing post‐operative pain management and opioid use from both patients' and health care professionals' perspectives using the TDF. It highlights the complex challenges in managing post‐operative pain while mitigating the risks of long‐term opioid use, underscoring the need to align patient and provider expectations. The findings emphasize the necessity for targeted interventions that address key barriers, such as continuity of care, pre‐operative education and communication gaps, while leveraging enablers like pharmacist involvement and digital tools to improve pain management and support safe opioid tapering.

By identifying critical BCTs and providing actionable recommendations, this study contributes to the future development of co‐designing interventions aimed at optimizing post‐operative pain management and reducing opioid‐related harms. However, to maximize the effectiveness of these interventions, further research is needed to explore their feasibility, acceptability and real‐world implementation in diverse health care settings. Ultimately, this research lays the groundwork for improving pain management practices and promoting safer opioid use across clinical practice, with the potential to enhance patient outcomes and address the growing concerns around opioid dependence and misuse.

## AUTHOR CONTRIBUTIONS

**Neetu Bansal:** Conceptualization; methodology; data curation; writing – review and editing; writing – original draft. **Rhiannon E. Hawkes:** Formal analysis; writing – review and editing. **Li‐Chia Chen:** Conceptualization; methodology; supervision; project administration; writing – review and editing. **Darren M. Ashcroft:** Conceptualization; methodology; supervision; writing – review and editing. **Christopher J. Armitage:** Conceptualization; methodology; supervision; formal analysis; writing – review and editing.

## CONFLICT OF INTEREST STATEMENT

All authors declare no conflict of interest.

## Supporting information


Data S1.

## Data Availability

The data that support the findings of this study are available from the corresponding author upon reasonable request.

## References

[bjhp70021-bib-0001] Ahmed, J. , Khan, S. , Lim, M. , Chandrasekaran, T. V. , & Macfie, J. (2012). Enhanced recovery after surgery protocols – Compliance and variations in practice during routine colorectal surgery. Colorectal Disease, 14, 1045–1051. 21985180 10.1111/j.1463-1318.2011.02856.x

[bjhp70021-bib-0002] Atkins, L. , Francis, J. , Islam, R. , O'connor, D. , Patey, A. , Ivers, N. , Foy, R. , Duncan, E. M. , Colquhoun, H. , Grimshaw, J. M. , Lawton, R. , & Michie, S. (2017). A guide to using the theoretical domains framework of behaviour change to investigate implementation problems. Implementation Science, 12, 77. 28637486 10.1186/s13012-017-0605-9 PMC5480145

[bjhp70021-bib-0003] Atkins, N. , & Mukhida, K. (2022). The relationship between patients' income and education and their access to pharmacological chronic pain management: A scoping review. Canadian Journal of Pain, 6, 142–170. 36092247 10.1080/24740527.2022.2104699 PMC9450907

[bjhp70021-bib-0004] Bansal, N. , Armitage, C. J. , Hawkes, R. E. , Tinsley, S. , Ashcroft, D. M. , & Chen, L.‐C. (2024). Decoding behaviour change techniques in opioid deprescribing strategies following major surgery: A systematic review of interventions to reduce postoperative opioid use. BMJ Quality and Safety, 34, 166–177. 10.1136/bmjqs-2024-017265 39074984

[bjhp70021-bib-0005] Bate, P. , & Robert, G. (2006). Experience‐based design: From redesigning the system around the patient to co‐designing services with the patient. Quality & Safety in Health Care, 15, 307–310. 17074863 10.1136/qshc.2005.016527 PMC2565809

[bjhp70021-bib-0007] Brummett, C. M. , Waljee, J. F. , Goesling, J. , Moser, S. , Lin, P. , Englesbe, M. J. , Bohnert, A. S. B. , Kheterpal, S. , & Nallamothu, B. K. (2017). New persistent opioid use after minor and major surgical procedures in US adults. JAMA Surgery, 152, e170504. 28403427 10.1001/jamasurg.2017.0504 PMC7050825

[bjhp70021-bib-0008] Cane, J. , O'connor, D. , & Michie, S. (2012). Validation of the theoretical domains framework for use in behaviour change and implementation research. Implementation Science, 7, 37. 22530986 10.1186/1748-5908-7-37 PMC3483008

[bjhp70021-bib-0009] Cho, H. E. , Billig, J. I. , Byrnes, M. E. , Nasser, J. S. , Kocheril, A. P. , Haase, S. C. , Waljee, J. F. , & Chung, K. C. (2021). A qualitative study of patient protection against postoperative opioid addiction: A thematic analysis of self‐agency. Plastic and Reconstructive Surgery, 147, 1124–1131. 33890894 10.1097/PRS.0000000000007841 PMC8074995

[bjhp70021-bib-0010] Collaborative, T. (2024). Patterns of opioid use after surgical discharge: A multicentre, prospective cohort study in 25 countries. Anaesthesia, 79, 924–936. 38721718 10.1111/anae.16297

[bjhp70021-bib-0011] Donetto, S. , Paola, P. , Vicki, T. , & Robert, G. (2015). Experience‐based co‐design and healthcare improvement: Realizing participatory design in the public sector. The Design Journal, 18, 227–248.

[bjhp70021-bib-0012] Friebel, R. , Yoo, K. J. , & Maynou, L. (2022). Opioid abuse and austerity: Evidence on health service use and mortality in England. Social Science & Medicine, 298, 114511. 34763968 10.1016/j.socscimed.2021.114511

[bjhp70021-bib-0013] Gale, N. K. , Heath, G. , Cameron, E. , Rashid, S. , & Redwood, S. (2013). Using the framework method for the analysis of qualitative data in multi‐disciplinary health research. BMC Medical Research Methodology, 13, 117. 24047204 10.1186/1471-2288-13-117 PMC3848812

[bjhp70021-bib-0014] Gan, T. J. (2017). Poorly controlled postoperative pain: Prevalence, consequences, and prevention. Journal of Pain Research, 10, 2287–2298. 29026331 10.2147/JPR.S144066 PMC5626380

[bjhp70021-bib-0015] Hinds, S. , Miller, J. , Maccani, M. , Patino, S. , Kaushal, S. , Rieck, H. , Walker, M. , Brummett, C. M. , Bicket, M. C. , & Waljee, J. F. (2022). Patient risk screening to improve transitions of care in surgical opioid prescribing: A qualitative study of provider perspectives. Regional Anesthesia and Pain Medicine, 47, 475. 35697386 10.1136/rapm-2021-103304 PMC9240329

[bjhp70021-bib-0016] Johnston, M. , Carey, R. N. , Connell Bohlen, L. E. , Johnston, D. W. , Rothman, A. J. , De Bruin, M. , Kelly, M. P. , Groarke, H. , & Michie, S. (2020). Development of an online tool for linking behavior change techniques and mechanisms of action based on triangulation of findings from literature synthesis and expert consensus. Translational Behavioral Medicine, 11, 1049–1065. 10.1093/tbm/ibaa050 PMC8158171 32749460

[bjhp70021-bib-0017] Jordan, M. , Latif, A. , Mullan, J. , & Chen, T. F. (2021). Opioid medicines management in primary care settings: A scoping review of quantitative studies of pharmacist activities. British Journal of Clinical Pharmacology, 87, 4504–4533. 34041786 10.1111/bcp.14915

[bjhp70021-bib-0018] Keyworth, C. , Epton, T. , Goldthorpe, J. , Calam, R. , & Armitage, C. J. (2019). It's difficult, I think it's complicated': Health care professionals' barriers and enablers to providing opportunistic behaviour change interventions during routine medical consultations. British Journal of Health Psychology, 24, 571–592. 30977291 10.1111/bjhp.12368 PMC6766974

[bjhp70021-bib-0019] Klueh, M. P. , Sloss, K. R. , Dossett, L. A. , Englesbe, M. J. , Waljee, J. F. , Brummett, C. M. , Lagisetty, P. A. , & Lee, J. S. (2019). Postoperative opioid prescribing is not my job: A qualitative analysis of care transitions. Surgery, 166, 744–751. 31303324 10.1016/j.surg.2019.05.033 PMC7068723

[bjhp70021-bib-0020] Kovačević, I. , Pavić, J. , Filipović, B. , Ozimec Vulinec, Š. , Ilić, B. , & Petek, D. (2024). Integrated approach to chronic pain‐the role of psychosocial factors and multidisciplinary treatment: A narrative review. International Journal of Environmental Research and Public Health, 21, 1135. 39338018 10.3390/ijerph21091135 PMC11431289

[bjhp70021-bib-0021] Kuntz, J. L. , Schneider, J. L. , Firemark, A. J. , Dickerson, J. F. , Papajorgji‐Taylor, D. , Reese, K. R. , Hamer, T. A. , Marsh, D. , Thorsness, L. A. , Sullivan, M. D. , Debar, L. L. , & Smith, D. H. (2020). A pharmacist‐led program to taper opioid use at Kaiser Permanente northwest: Rationale, design, and evaluation. The Permanente Journal, 24, 19.216. 33196429 10.7812/TPP/19.216 PMC7213382

[bjhp70021-bib-0022] Magee, M. R. , Gholamrezaei, A. , Mcneilage, A. G. , Sim, A. , Dwyer, L. , Ferreira, M. L. , Darnall, B. D. , Glare, P. , & ASHTON‐James, C. E. (2022). A digital video and text messaging intervention to support people with chronic pain during opioid tapering: Content development using co‐design. JMIR Formative Research, 6, e40507. 36355415 10.2196/40507 PMC9693745

[bjhp70021-bib-0023] Michie, S. , Van Stralen, M. M. , & West, R. (2011). The behaviour change wheel: A new method for characterising and designing behaviour change interventions. Implementation Science, 6, 42. 21513547 10.1186/1748-5908-6-42 PMC3096582

[bjhp70021-bib-0024] Myles, P. S. , & Bui, T. (2022). Opioid‐free analgesia after surgery. The Lancet, 399, 2245–2247. 10.1016/S0140-6736(22)00777-2 35717974

[bjhp70021-bib-0025] Roberts, A. O. , & Richards, G. C. (2023). Is England facing an opioid epidemic? British Journal of Pain, 17, 320–324. 37342398 10.1177/20494637231160684 PMC10278447

[bjhp70021-bib-0026] Simpson, J. C. , Bao, X. , & Agarwala, A. (2019). Pain management in enhanced recovery after surgery (ERAS) protocols. Clinics in Colon and Rectal Surgery, 32, 121–128. 30833861 10.1055/s-0038-1676477 PMC6395101

[bjhp70021-bib-0027] Soneji, N. , Clarke, H. A. , Ko, D. T. , & Wijeysundera, D. N. (2016). Risks of developing persistent opioid use after major surgery. JAMA Surgery, 151, 1083–1084. 27533746 10.1001/jamasurg.2016.1681

[bjhp70021-bib-0028] Varrassi, G. , Muzammil, M. A. , Kumar, S. , Rekatsina, M. , Moka, E. , Paladini, A. , & Khatri, M. (2023). #36889 closing the gaps in postoperative pain management: Challenges and future perspectives. Regional Anesthesia and Pain Medicine, 48, A355.

[bjhp70021-bib-0029] Walton, L. L. , Duff, E. , Arora, R. C. , & Mcmillan, D. E. (2023). Surgery patients' perspectives of their role in postoperative pain: A scoping review. International Journal of Nursing Studies Advances, 5, 100124. 38746556 10.1016/j.ijnsa.2023.100124 PMC11080476

