# Supplementary material for: Perceptions of patients and health care professionals on postoperative pain management: Key factors influencing persistent opioid use
Source: Br J Health Psychol. 2025 Sep 11;30(3):e70021. doi: 10.1111/bjhp.70021 (PMC12423896; doi:10.1111/bjhp.70021)
Supplement: Supplementary file 1 — Data S1. [file BJHP-30-0-s001.docx]

# Appendices

Appendix S1. Theoretical domains framework domain labels and definitions

| **Domain** | **Definition** |
| --- | --- |
| 1. Knowledge | An awareness of the existence of something. |
| 2. Skills | An ability or proficiency acquired through practice. |
| 3. Social/Professional Role and Identity | A coherent set of behaviours and displayed personal qualities of an individual in a social or work setting. |
| 4. Beliefs about Capabilities | Acceptance of the truth, reality, or validity about an ability, talent, or facility that a person can put to constructive use. |
| 5. Optimism | The confidence that things will happen for the best or that desired goals will be attained. |
| 6.Beliefs about Consequences | Acceptance of the truth, reality, or validity about outcomes of a behaviour in a given situation. |
| 7. Reinforcement | Increasing the probability of a response by arranging a dependent relationship, or contingency, between the response and a given stimulus. |
| 8. Intentions | A conscious decision to perform a behaviour or a resolve to act in a certain way. |
| 9. Goals | Mental representations of outcomes or end states that an individual wants to achieve. |
| 10. Memory, Attention and Decision Processes | The ability to retain information, focus selectively on aspects of the environment and choose between two or more alternatives. |
| 11. Environmental Context and Resources | Any circumstance of a person's situation or environment that discourages or encourages the development of skills and abilities, independence, social competence, and adaptive behaviour. |
| 12. Social influences | Those interpersonal processes that can cause individuals to change their thoughts, feelings, or behaviours. |
| 13. Emotion | A complex reaction pattern, involving experiential, behavioural, and physiological elements, by which the individual attempts to deal with a personally significant matter or event. |
| 14. Behavioural Regulation | Anything aimed at managing or changing objectively observed or measured actions. |

(Note) The definitions were based on Cane *et al.* 2012.

Appendix S2. Focus group schedule for patient participants

**Developing an intervention to optimise acute pain and opioid use after surgery**

**Opening**

(Introduction, briefing the purposes of the focus group, set up the ground rules)

(Aim of this meeting)

We've invited you because your experiences with post-surgical pain management are precious. Today, we want to hear about your experiences and views of surgical pain management, especially with using potent pain relievers, such as opioids, and discuss how we can better support patients facing challenges with their surgical pain and pain medications. We aim to develop practical strategies to improve how to handle pain and pain medication issues for patients after painful surgeries.

(Schedule and housekeeping)

This focus group discussion is scheduled for 120 minutes. We have prepared tea/coffee, and refreshments for you during the meeting and lunch after the meeting. Restrooms are located [provide directions]. In an emergency, fire exits are located [provide directions].

(Confidentiality and anonymity of recording)

During our focus group discussion, we will facilitate the conversation, take notes and audio-record the discussion. We will ensure all the conversations recorded will be kept confidential and anonymous. Your honesty is crucial, and there are no right or wrong answers.

(Ground rules)

Before we begin, let's agree on some ground rules:

We kindly ask that we take turns speaking to ensure a clear audio recording and allow others to express their opinions even if you disagree with them.

If you want to share your views, please raise your hand when someone else is done speaking, and please respect the confidentiality of the discussion.

Thank you for being a part of this vital conversation. Your insights will make a real difference.

1. **Ice breaking**

Before we start, can you briefly introduce yourselves, stating your names, what painful surgery you had in the past, and what kind of pain management strategies you revived, including medicines?

(Fact findings: to explore patients' pain management experiences after surgery and their initial pain management.)

**Prompt questions:**

- How was your pain managed after surgery?
- Which painkillers were prescribed to you?
- What are the critical moments/emotions/challenges you faced?
- What coping mechanisms have you used to manage post-surgery pain?
- Can you share any helpful personal strategies or support from healthcare professionals?

1. **About medicine information and communication with healthcare providers**

If you had experience in using opioids or pain medication for managing surgical pain, I would like to know more about what information was provided to you.

**Prompt questions:**

- Did you feel adequately informed about the risks and benefits of opioid use?
- Did you experience any side effects with the pain medicines?
- Were you satisfied with the pain management strategies, including the use of opioids?
- Did you discuss pain medication with doctors/pharmacists/nurses or your GP/practice pharmacist after discharge?
- Can you identify any potential improvement in the care you received?

(To capture brief insights and suggestions for enhancing pain management services.)

- If yes, how did you feel about the information provided during these discussions?
- What additional information would you have liked to receive regarding pain medications and their potential side effects?

1. **About stopping the pain medicines**

If you had experiences in using opioids or pain medication, when and how did you reduce or stop taking these medications?

(To identify enablers, barriers, and appropriate strategies to tapering opioids)

**Prompt questions:**

- Did you utilise any alternative therapies or pain management techniques during this process (explore non-opioid drug options, non-pharmacological therapy)?
- What factors enabled or hindered your tapering of opioids?

(To identify any critical moments/emotions/challenges encountered during the tapering process and strategies to overcome them.)

- In your experience, what are the most effective strategies for tapering opioid use?

(To gain an understanding of various coping mechanisms patients use to manage post-surgery pain.)

- How do you believe healthcare professionals can better support patients in tapering opioids effectively?

(To highlight personal strategies and support from healthcare professionals, identify potential improvements, and capture brief insights and suggestions for enhancing pain management services.)

1. **About the potential interventions**

Based on the discussion so far, what should be changed in the future to help patients handle pain and pain medication after painful surgeries?

Our research team is developing a tool called "eTAPER", a reminder built on GP's prescribing records to remind GPs or GP-based practice pharmacists that the patient is still on opioids or pain medication after surgery. It allows GPs to assess patients' pain after surgery, review the need for ongoing pain medication, and discuss appropriate tapering with the patients. We hope to co-deign the features with patients and healthcare professionals to ensure the appropriate actions will be taken in response to the reminder.

How do you feel about this tool?

**Prompt questions:**

- Do you think this will be helpful?
- If so, how would you like GPs or practice pharmacists to use or respond to this reminder?
- What features should be included in the tool to help with the tapering of opioids?

(To engage patients and healthcare professionals in discussing potential improvements, capture brief insights and suggestions for enhancing pain management services)

**Closing**

As we wrap up today's discussion, let's recap some key points we've covered [briefly mention a few key discussion points]. Did we miss anything important, or does anyone have additional thoughts to share? All input is invaluable.

A big thank you to each of you for your active participation and contributions today. Your input will be essential as we analyse the responses and draw conclusions.

The following sessions will be held online, and you will have opportunities to co-design the tool's features and recommend actions with healthcare professionals during the following two sessions. Your involvement can continue to shape and improve healthcare for more people. Feel free to contact me if you have any questions about future events.

For today's session, please help me to complete an expense form. Lunch will be ready at 12:00. Before we finish today's session, I will interview you individually for two key themes that emerge from today's focus group. This interview will be video-recorded to produce a trigger film to help a focus group discussion with healthcare professionals.

Thank you very much.

Appendix S3. Focus group schedule for healthcare professional participants

**Developing an intervention to optimise acute pain and opioid use after surgery**

**Opening**

(Introduction, briefing the purposes of the focus group, set up the ground rules)

Good morning, everyone. Welcome to today's focus group meeting. My name is Neetu Bansal, and I am a pharmacist researcher at the University of Manchester. Today, we are here to develop a supporting tool for pain management. I will facilitate this session, which will also be supported by Leanne Hill, Fiona Angus, Wan-Chuen Liao, and Li-Chia Chen.

(Aim of this meeting)

- We've invited you because of your experiences in managing patients' post-surgical pain, which can provide vital insights in shaping the eTAPER tool, a web-based audit and feedback tool aimed at identifying patients' prescribed opioids in primary care after surgery.
- I have some slides to share on our recent work illustrating the motivation and background in developing the eTAPER tool (present three slides).
- Today, we want to hear your views and opinions on the challenges in surgical pain management, especially with using potent analgesics, such as opioids, and discuss how we can better support patients facing challenges with their surgical pain and pain medications.
- At the end of today's session, we aim to identify practical strategies to improve the handling of pain and pain medication issues for patients after surgery so that we can further develop in the next co-design focus group with patients.

(Schedule and housekeeping)

- This focus group discussion is scheduled for around 2 hours.
- Facilitators will help with the focus group discussions, take notes and manage the time.
- If you need a break during the discussion, please inform facilitators using the chat box.
- The facilitator will remain impartial throughout the event.

[To facilitate effective conversations, we will divide into two breakout rooms, each led by a facilitator.]

(Confidentiality and anonymity of recording)

- Throughout our discussion, we will be recording audio.
- Your honesty is paramount, and there are no right or wrong answers. Please be assured all conversations will be treated with utmost confidentiality and anonymity. Do not disclose identifiable information outside this focus group.

(Ground rules)

Before we begin, let's agree on some ground rules:

- We kindly ask that we take turns speaking to ensure a clear audio recording and allow others to express their opinions even if you disagree with them.
- If you want to share your views, please use the '**raise your hand**' function and respect the discussion's confidentiality.
- Please also express your views **verbally** to enable audio recording.

Thank you for being a part of this vital conversation. Your insights will make a real difference.

**Ice breaking**

Before we start, could you please take a moment to introduce yourself, including your name, speciality, and current role?

(Ask this question before splitting into the breakout rooms to ensure the group knows each other.)

Thank you. Now, we will split into two breakout rooms for the main discussion for [60 minutes], and then we will get back together in the main room at [11:40] to wrap up our discussions today. I will also highlight the activities for our subsequent focus group sessions. See you back at [11:40].

Hello, and welcome to the discussion of this breakout room. My name is [X], and I will facilitate the discussions. Together with me is [X] to help with this session. We have [X, X, X, X, X, and X] (name the participants) in this group.

1. **About healthcare providers' perceived challenges in managing post-surgical pain**

To start our discussion, could you share some of your experiences in managing patients' post-surgical pain and what challenges you encountered?

(Fact findings: To gather insights on healthcare professionals' experiences in post-surgical pain management and their encounters with patients prescribed opioids for pain relief.)

**Prompt questions:**

- Do you think what were *the roles of opioid* medication?
- What are your views on the *effectiveness of opioid medication in managing post-surgical pain*? In what situations do you find opioids to be most beneficial?
- Have you noticed any trends or patterns in the prescription of opioids for post-surgical pain? Are there any *factors that influence your decision to prescribe* or not?

1. **About medicine information and communication with healthcare providers**

According to your experience in prescribing or reviewing patients' opioids or pain medication for managing surgical pain, I would like to know more about what information you provide to patients.

**Prompt questions:**

- Did you feel patients are adequately informed about the risks and benefits of opioid use?
- Can you share any experiences you've had with patients who have been prescribed opioids for post-surgical pain?

How do you *monitor their usage* and *address any concerns or side effects* they may experience?

- What *resources or support systems* do you rely on for guidance in managing post-surgical pain and opioid prescribing practices?
- *How do you approach patient education and communication* regarding the use of opioids for post-surgical pain?

*What information* do you typically provide patients to ensure safe and effective use?

- *What non-opioid alternatives* do you typically consider for managing post-surgical pain?

How do you incorporate these alternatives into your practice?

(To gather concise insights and suggestions for enhancing pain management services.)

1. **About stopping the pain medicines**

Regarding deprescribing/tapering opioids or pain medication, can you share when and how did you reduce these medications?

(To identify enablers, barriers, and appropriate strategies to tapering opioids)

**Prompt questions:**

- Could you share *any challenges or barriers* you've encountered when tapering opioids or pain medications?
- What *factors influence your decision to taper* opioids or other pain medications for patients?
- Did you utilise *any alternative therapies* or *pain management techniques* during this process (explore non-opioid drug options, non-pharmacological therapy)?
- What *strategies* or *protocols* do you find effective in safely tapering opioids or other pain medications?
- *How do you monitor* patients during tapering to ensure their safety and comfort?
- Are there any *specific guidelines or resources* you rely on when tapering opioids or other pain medications?

(These questions aim to explore experiences, challenges, and best practices related to tapering opioids and other pain medications.)

1. **About the potential interventions**

As healthcare professionals, based on our discussion, **what changes do you believe should be implemented in the future** to assist patients in better managing pain and pain medication post-surgery?

| Our research team is developing a tool called "eTAPER", a reminder built on GP's prescribing records to remind GPs or GP-based practice pharmacists that the patient is still on opioids or pain medication after surgery.  This reminder can trigger healthcare professionals to assess patient's pain after surgery, review the need for ongoing pain medication, and discuss appropriate tapering with the patients.  We hope to co-design the features with patients and healthcare professionals to ensure the appropriate actions will be taken in response to the reminder. |
| --- |

What are your thoughts on the concept and potential effectiveness of the tool?

**Prompt questions:**

- How do you foresee the integration of the eTAPER tool into your current practice? Are there *any specific challenges or barriers* you anticipate in its implementation?
- How do you think patients would respond to having their opioid medication tapered as a result of the eTAPER tool? (perceived value/benefit/usefulness)
- *What additional support or resources* do you believe would be beneficial for healthcare professionals using the eTAPER tool to facilitate discussions about pain management and tapering with opioids?
- *What features* should be included in the tool to help with the tapering of opioids?

(To engage healthcare professionals in discussing potential improvements, capture brief insights and suggestions for enhancing pain management services)

**Closing**

Thank you for rejoining the main group. I hope you had an insightful discussion in your breakout room.

As we wrap up today's discussion, let's recap some key points we've covered [briefly mention a few key discussion points]. Did we miss anything important, or does anyone have additional thoughts to share? All input is invaluable.

A big thank you to each of you for your active participation and contributions today. Your input will be essential as we analyse the responses and draw conclusions.

I will email an expense form to complete for your time on the study and subsequent sessions.

The following sessions will be held online, and you will have opportunities to co-design the tool's features and recommend actions to patients during the following two sessions. Your involvement can continue to shape and improve healthcare for more people.

Thank you very much. See you at the next session at 10:00-12:00 on Tuesday, 23 April 2024.

Appendix S4. Summary of the behaviour change techniques identified from included studies

| **Group** | **Behaviour Change Techniques** | **Definition** |
| --- | --- | --- |
| 2. Feedback and monitoring | 2.3 Self-monitoring of behaviour | Establish a method for the person to monitor and record their behaviours(s) as part of a behaviour change strategy |
| 3. Social support | 3.2 Social support (practical) | Advise on, arrange, or provide practical help *(e.g. from friends, relatives, colleagues, 'buddies' or staff)* for the performance of the behaviour. |
| 4. Shaping knowledge | 4.1 Instruction on how to perform the behaviour | Advise or agree on how to perform the behaviour (includes 'Skills training') |
| 5. Natural consequences | 5.1 Information about health consequences | Provide information (e.g. *written, verbal, visual*) about the health consequences of performing the behaviour. |
| 7. Associations | 7.1 Prompts/cues | Introduce or define environmental or social stimulus to prompt or cue the behaviour. The prompt or cue would typically occur at the time or place of performance. |
| 8. Repetition and substitution | 8.7 Graded tasks | Set easy-to-perform tasks, making them increasingly difficult but achievable until the behaviour is performed. |
| 11. Regulation | 11.2 Reduce negative emotions | Advise on ways of reducing negative emotions to facilitate performance of the behaviour |
| 12. Antecedents | 12.1 Restructuring the physical environment | Change, or advise to change the physical environment in order to facilitate performance of the wanted behaviour or create barriers to the unwanted behaviour (other than prompts/cues, rewards and  punishments) |
